# The association between arterial stiffness and cancer occurrence: Data from Kailuan cohort study

**DOI:** 10.3389/fcvm.2023.1112047

**Published:** 2023-03-01

**Authors:** Yinong Jiang, Aijun Xing, Tesfaldet Habtemariam Hidru, Jiatian Li, Xiaolei Yang, Shuohua Chen, Yun-Long Xia, Shouling Wu

**Affiliations:** ^1^Department of Cardiology, Institute of Cardiovascular Diseases, First Affiliated Hospital of Dalian Medical University, Dalian, Liaoning, China; ^2^Department of Cardiology, Kailuan General Hospital, North China University of Science and Technology, Tangshan, Hebei, China; ^3^Health Department of Kailuan Group, Tangshan, China

**Keywords:** arterial stiffness, malignancy, mortality, cancer, brachial-ankle pulse wave velocity

## Abstract

**Background:**

This study aimed to investigate whether increased arterial stiffness, measured by brachial-ankle pulse wave velocity (baPWV) is associated with cancer.

**Materials and methods:**

A total of 45,627 Chinese adults underwent a baPWV examination. The participants were followed up from 1st January 2012 to 31st December 2018. Cox proportional hazards model was used to assess the association between the baPWV values and cancer.

**Results:**

During a total follow-up duration of 172,775.69 person-years, there were 553 new cases of cancer. The subjects in the highest baPWV group showed an increased risk of cancer when compared with the lowest baPWV group as confirmed by multivariate-adjusted hazard ratios (HR = 1.51, 95% CI = 1.14∼2.00) in the entire cohort. Compared with participants in the lowest baPWV group, the HRs (95% CI) for digestive cancer in the second and third groups were 1.55 (1.00∼2.40) and 1.99 (1.19∼3.33), respectively. The Kaplan-Meier analysis demonstrated a significant increase in cancer in participants with a baPWV ≥ 18 m/s (*P* < 0.001). Compared with the lowest baPWV group, the highest baPWV group showed an increased risk of cancer in men (HR = 1.72, 95% CI = 1.22∼2.43) and those < 60 years (HR = 1.75, 95% CI = 1.20∼2.55), respectively.

**Conclusion:**

Increased arterial stiffness measured by baPWV is associated with cancer occurrence, especially digestive cancer occurrence.

**Clinical trial registration:**

ClinicalTrials.gov, identifier ChiCTR-TNRC-11001489.

## 1. Introduction

The risk of arterial stiffness increases following anticancer therapies like radiotherapy or chemotherapy (1, 2). Newer studies have suggested that the association between cardiovascular diseases and cancer extends beyond the toxicities that occur during cancer treatment (3, 4). Recently, heart failure (HF) was identified to have potentially increased the likelihood of cancer independent of other risk factors (5–7). Overall, cancer and cardiovascular diseases are public health and economic concerns. Worldwide, an estimated 19.3 million new cancer cases and almost 10.0 million cancer deaths occurred in 2020 (8). Globally, the prominence in the prevalence of cardiovascular diseases and cancer is partly attributed to poor adherence to a healthy lifestyle (9). Thus, early detection or prediction of cancer is important from the perspective that cancer and cardiovascular disease are the leading cause of mortality, disability, and global healthcare costs.

Arterial stiffness describes the hardening of the arterial system and is a surrogate marker for impaired vascular function and structure. The development of arterial stiffness is a highly integrated process, connecting multiple contributors such as inflammation, immune cell dysfunction, and extracellular matrix remodeling (10, 11). These factors are not only important in the development of arterial stiffness but also play meaningful roles in the pathogenesis of cancer.

A recent large cohort study by Harding et al. has found that hypertension is associated with an increased risk of cancer incidence and mortality, which has also been supported by several animal hypertension models (12, 13). Further, recent research confirmed that early cardiac remodeling promoted tumor growth and metastasis (14). However, hypertension is typically an advanced manifestation of arterial remodeling. It is important to detect whether any earlier vascular changes have already been associated with cancer. One method to assess early vascular remodeling is brachial-ankle pulse wave velocity (baPWV), a technique that measures systemic arterial stiffness (10, 15). Whether high baPWV is associated with cancer risk in the general population remains uncovered. Therefore, the purpose of this study was to investigate the association between arterial stiffness status (measured by baPWV) and cancer occurrence.

## 2. Materials and methods

### 2.1. Study design

**FIGURE 1:**
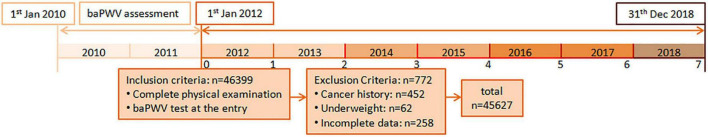
The flow chart of this large cohort study. Participants who received baseline baPWV with biennial physical examinations were eligible for this study. The baseline baPWV was assessed from January 2010 to December 2011. Participants were followed up for cancer occurrence between January 2012 and December 2018. baPWV, Brachial-ankle pulse wave velocity.

This is an ancillary study of the Kailuan cohort study conducted to explore the relationship between different categories of arterial stiffness and cancer occurrence. The Kailuan study is an ongoing prospective cohort (clinical trial number ChiCTR-TNRC-11001489) that recruits adult participants from Tangshan City, China. The details regarding the Kailuan cohort study design have been reported in other studies (16, 17). Briefly, this ancillary study recruited a total of 46,399 adult participants between January 2010 and December 2011 from 11 hospitals affiliated with the Kailuan Group. The cohort administered questionnaire assessment and underwent clinical and laboratory examinations upon enrollment every 2 years till December 2018 (17). We measured baPWV, a simple and non-invasive examination, to determine arterial stiffness (10, 16). The baseline baPWV was assessed from 1st January 2010 to 31st December 2011. Participants were followed up for cancer occurrence between January 2012 and December 2018 (Figure 1).

### 2.2. Population

In the current study, 46,399 Chinese participants were included after satisfying the requirements of a minimum age of 18 years and a complete physical examination with a baPWV test. Participants with the following criteria were then excluded from the study: those with a cancer diagnosis prior to baseline (*n* = 452), those who were underweight at baseline (*n* = 62), and those who had incomplete data (*n* = 258). A total of 45,627 participants were included in the final data analysis after exclusion criteria were applied. The study protocol was approved by the institutional review board of the Kailuan General Hospital. All procedures were performed in line with the declaration of Helsinki and its amendments, and all participants provided written informed consent to participate in this study.

### 2.3. Assessment of the arterial stiffness

Arterial stiffness was evaluated between January 2010 and December 2011 with baPWV conducted by a networked arteriosclerosis detection system, BP-203 RPE III [Omron Health Medical (China), Co., Ltd.]. All baPWV measurements were carried out by qualified physicians and nurses and the measurements were recorded from 7 AM to 9 AM following the manufacturer’s instructions. Prior to baPWV measurement, participants were prohibited from smoking and consuming caffeinated beverages or alcohol for at least 3 h and were restricted from engaging in exercise for at least 30 min. Participants were then seated in a room with temperature regulated between 22 and 25^°^C for at least 5 min and subsequently instructed to lie down on the examination table in a supine position with the device cuffs bound to both their arms and legs. The lower edge of the arm cuff was placed 2–3 cm above the cubital fossa transverse striation, while the lower edge of the ankle cuff was adjusted 1–2 cm above the superior edge of the medial malleolus. The electrocardiogram electrodes were attached to bilateral wrists, and a microphone was mounted on the left side of the sternum to detect heart sounds. The baPWV measurement was performed by physicians. As baPWV measurement is an operator-dependent procedure, the baPWV readings were measured twice to avoid measurement error, and the mean of the two baPWV readings was obtained and recorded as baPWV values. Additional measurements were made if there was a difference of > 2 m/s following the first two readings.

### 2.4. Blood pressure measurement

We simultaneously measured the blood pressure (BP) in four extremities in the supine position after at least 5 min bed rest. We defined inter-arm BP difference (IAD) as the absolute difference of systolic BPs measured in the right and left arms. The ankle-brachial index (ABI) was calculated as the ratio of ankle systolic BP to brachial systolic BP (a higher value) for both legs, and the lower ABI value was used in the subsequent analysis. The lower ABI and/or the larger IAD suggest the presence of peripheral artery disease (PAD) (18, 19).

### 2.5. Other measurements

Every patient performed a routine medical check-up every 2 years following baPWV measurement. Also, a structured interview was conducted at each examination to obtain data on demographic and clinical characteristics which comprised lifestyle information, family background, and the use of any medications. Blood specimens were taken (fasting > 8 h) and were biochemically analyzed for the level of C-reactive protein (CRP) and fasting plasma glucose (FPG). Standard enzymatic processes were carried out to detect the concentration of high-density lipoprotein (HDL) and serum total cholesterol (TC). All the above indexes were measured by HITACHI AUTOMATIC ANALYZER 3110 [Hitachi Instrument (Suzhou), Ltd.]. Hypertension was characterized as systolic BP (SBP) ≥ 140 mmHg and/or diastolic BP (DBP) ≥ 90 mmHg or a self-reported history of hypertension with the current use of antihypertensive drugs. Diabetes mellitus was defined as FPG ≥ 7.0 mmol/L or a self-reported history of diabetes mellitus and current diabetes treatment. Smoking status was classified into three groups: never-smoker, a former smoker, and current smoker, while physical exercise was divided into two categories: high/intense activity if participants reported exercise for ≥ 4 h per week and sedentary/moderate activity if participants reported physical activity for < 4 h per week. Hepatic dysfunction was clinically diagnosed by physicians according to the guidelines for liver diseases. Hyperbilirubinemia, an increase in serum transaminases, alkaline phosphatase (AP), and γ glutamyl-transferase (GGT), and a decrease in serum albumin and coagulation factors levels are the main laboratory parameters on which the diagnosis of hepatic dysfunction is based (20–22).

### 2.6. Outcome assessment

All cancer events were confirmed from hospital diagnosis records and were identified using the International Classification of Diseases-10 (ICD-10) codes. In this study, we analyzed several categories of cancer, which included the digestive system (C15-C26), respiratory system (C34 + C39), urogenital system (C50-C58 for females, C60-C63 for males), and other systems (all codes beginning with C). The study participants were contacted periodically during follow-up and the cancer occurrence was confirmed through hospital records.

### 2.7. Statistical analysis

Similar to previously published studies, baPWV was categorized into 3 groups: baPWV < 14.0 m/s (normal elastic artery status), 14.0 ≤ baPWV < 18.0 m/s (elasticity decreasing status), and ≥ 18.0 m/s (arterial stiffness) (10). All continuous variables were normally distributed and expressed as mean ± SD, and categorical variables were expressed as counts and percentiles. Groups were tested for differences using the χ2 test and ANOVA for categorical and continuous data respectively.

Log-rank tests for trend and Kaplan-Meier methods were used to compare the distributions of cancer incidence and investigate the differences in survival as stratified by baPWV, respectively. For the outcomes of cancer incidence, two distinct Cox models were employed to look for possible associations with baPWV independently. The Cox proportional hazard model for our study was then employed to explore the association of 1 SD increase in baPWV (for each baPWV group), modeled as a continuous variable, with the occurrence of cancer. To account for potential confounding effects, we selected variables with *P* < 0.05 in the univariate Cox analyses or clinically associated with cancer occurrence were candidates for the multivariate Cox regression analysis. Thus, the multivariate Cox models were adjusted for age, gender, body mass index, mean arterial pressure, fasting plasma glucose, hepatic dysfunction, eGFR, anemia, uric acid, cigarette smoking, alcohol consumption, physical activity, C-reactive protein, TC, antihypertensive medication, HBsAg status, and family history of cancer. The proportional hazard assumption in the Cox model was tested and satisfied in all cases using the Schoenfeld residuals (Supplementary Figure 1). Furthermore, to investigate the pattern of quantitatively assessed baPWV associated with cancer occurrence, we analyzed the restricted spline curve. HRs of restricted cubic spline transformation of baPWV with 3 knots (5, 50, and 95% of baPWV value) and 50% of baPWV value as a reference were plotted.

The analysis was repeated using a fully adjusted model to test for the robustness of estimates in those who had no history of CVD (stroke and cardiac infarction), or those who had no sign of peripheral arterial disease (ABI ≤ 0.9 and/or IAD ≥ 15 mmHg). A *P* value of ≤ 0.01 was considered statistically significant. All statistical analyses were conducted using SAS 9.3 (SAS Institute; Cray, NC, USA).

## 3. Results

### 3.1. Baseline characteristics of the participants

During a median follow-up duration of 3.35 (interquartile range: 1.66–6.07) years, there were 553 new cases of cancer. All cancer patients were different; thus 1 cancer diagnosis was referred to 1 patient. Out of these new cases, 170 had cancer of the digestive system, 138 had cancer of the respiratory system, 89 had cancer of the urogenital system, and 156 had cancer of other systems. The mean (SD) ages of participants present in the baPWV < 14 m/s (*n* = 19,679), baPWV 14 m/s∼18 m/s (*n* = 17,720), and baPWV ≥ 18 m/s (*n* = 8,228) group were 41.46 ± 9.72, 50.40 ± 10.86, and 61.06 ± 11.51, respectively. Participants in higher groups of baPWV had higher mean SBP, DBP, and fasting plasma glucose than in lower groups. Additionally, participants in higher groups of baPWV were more likely to have hypertension. The demographic data for the study participants are shown in Table 1. The results of the univariate and multivariate logistic analysis are presented in Supplementary Table 1.

**TABLE 1:** Baseline clinical characteristics of participants.

Variables	baPWV < 14.0 m/s (*n* = 19,679)	14.0 ≤ baPWV < 18.0 m/s (*n* = 17,720)	baPWV ≥ 18.0 m/s (*n* = 8,228)	*P*-value
Age (year)	41.46 ± 9.72	50.40 ± 10.86	61.06 ± 11.51	<0.001
Male, n (%)	11600 (58.95)	14480 (81.72)	6767 (82.24)	<0.001
SBP (mmHg)	120.93 ± 14.60	135.14 ± 16.54	147.75 ± 19.54	<0.001
DBP (mmHg)	77.43 ± 9.58	84.69 ± 10.36	87.13 ± 11.47	<0.001
MAP (mmHg)	91.93 ± 10.40	101.50 ± 11.24	107.34 ± 12.30	<0.001
BMI (kg/m^2^)	24.46 ± 3.19	25.35 ± 3.06	25.26 ± 3.04	<0.001
hs-CRP > 2 mg/L (%)	4052 (8.88)	4807 (10.54)	2730 (5.98)	<0.001
TC (mmol/L)	4.77 ± 1.35	5.07 ± 1.46	5.14 ± 1.92	<0.001
HDL (mmol/L)	1.47 ± 0.67	1.44 ± 0.70	1.49 ± 0.82	<0.001
FPG (mmol/L)	5.30 ± 1.08	5.96 ± 1.84	6.80 ± 2.47	<0.001
Uric acid (mmol/L)	303.15 ± 96.00	326.84 ± 99.17	329.05 ± 95.51	<0.001
eGFR (mL/min*1.73 m^2^)	102.43 ± 23.40	96.86 ± 22.50	89.36 ± 20.65	<0.001
Current smoker, n (%)	5329 (27.04)	6303 (35.57)	2388 (29.02)	<0.001
Current drinker, n (%)	654 (3.32)	1021 (5.76)	503 (6.11)	<0.001
High/Intensive activity, n (%)	10355 (52.62)	9396 (53.02)	4490 (54.57)	0.011
Hepatic dysfunction, n (%)	1663 (8.45)	2004 (11.31)	746 (9.07)	<0.001
Anemia, n (%)	1315 (6.68)	862 (4.86)	334 (4.06)	<0.001
HBsAg positive, n (%)	188 (0.96)	146 (0.82)	65 (0.79)	0.262
Antihypertensive use, n (%)	785 (3.99)	2704 (15.26)	2467 (29.98)	<0.001
Tumor family history, n (%)	588 (2.99)	848 (4.79)	666 (8.09)	<0.001

BMI, body mass index; DBP, diastolic blood pressure; eGFR, estimated glomerular filtration rate; FPG, fasting plasma glucose; HBsAg, hepatitis B surface antigen; HDL-C, high density lipoprotein cholesterol; hs-CRP, high sensitivity C-reactive protein; MAP, mean arterial pressure; SBP, systolic blood pressure; TC, total cholesterol.

### 3.2. Cancer occurrence

**FIGURE 2:**
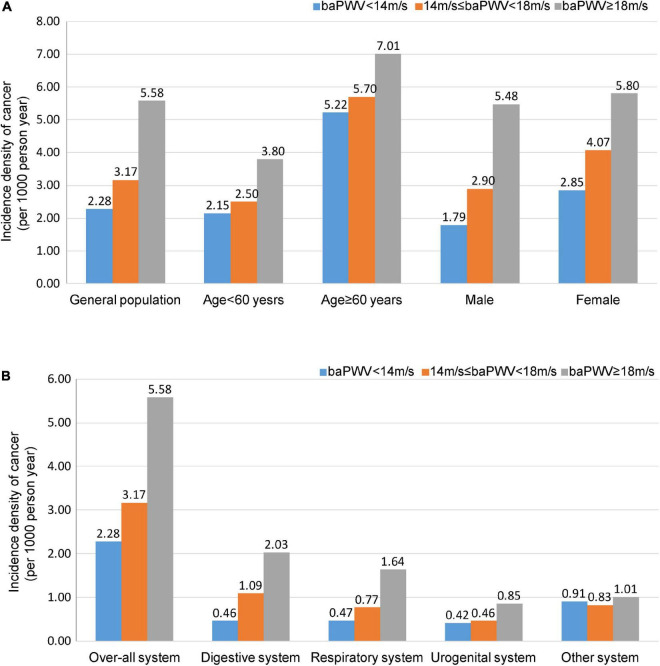
Incidence density of cancer. Participants are categorized into three groups: baPWV < 14.0 m/s, 14.0 ≤ baPWV < 18.0 m/s, and ≥ 18.0 m/s. Incidence density of cancer in the general population, men, women, individuals < 60 years old, and individuals ≥ 60 years old. **(A)** Incidence density of cancer in the different systems. **(B)** Values are presented as per 1,000-person year. baPWV, Brachial-ankle pulse wave velocity.

The incidence density of cancer occurrences increased across the different values of baPWV. The incidence density in the entire cohort increased from 2.28 per 1,000-person year in patients marginalized at baPWV < 14.0 m/s to 3.17 per 1,000-person year in patients with 14.0 ≤ baPWV < 18.0 m/s, and 5.58 per 1,000-person year in patients with baPWV ≥ 18.0 m/s (Figure 2A). Similarly, the incidence density shows a progressively higher risk of digestive cancer across normal to advanced arterial stiffness status (Figure 2B). Cumulative incidence of cancer in participants by groups of baPWV is showed in Supplementary Table 2.

### 3.3. Relationship between the baPWV and cancer occurrence

**FIGURE 3:**
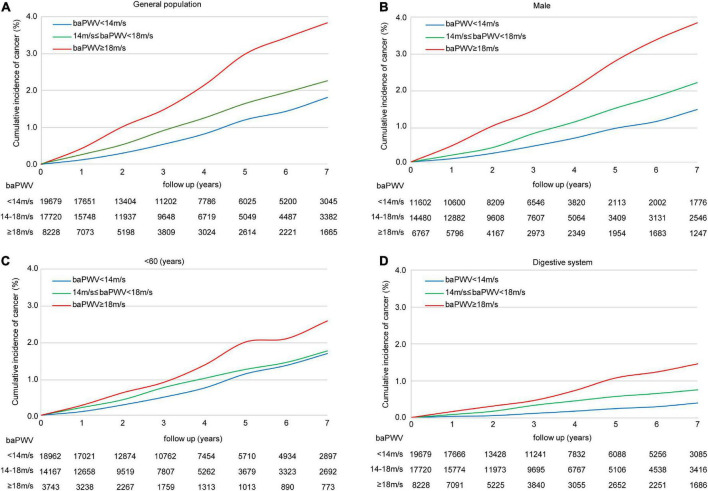
The Kaplan-Meier survival curves for cancer incidence. Participants are categorized into three groups according to baPWV values. The Kaplan-Meier survival curves demonstrate a significant increase in cancer incidence in the entire cohort **(A)** male **(B)** and participants <60 years **(C)** with a baPWV ≥ 18 m/s (all *P* < 0.001). The Kaplan-Meier survival curves demonstrate a significant increase in digestive cancer incidence in the entire cohort **(D)** with a baPWV ≥ 18 m/s (all *P* < 0.001). baPWV, Brachial-ankle pulse wave velocity.

The association between the baPWV and the risk of cancer is presented in Table 2 and Supplementary Table 3. The participants in the highest baPWV group had a significantly increased risk of cancer. Compared to participants in the lowest baPWV group, the HRs (95% CI) for cancer in the second and third groups were 1.16 (0.92∼1.46) and 1.51 (1.14∼2.00), respectively (*P* for trend = 0.004). With 1-SD increase in baPWV, the risk of cancer consistently increased (HR = 1.16, 95% CI: 1.05∼1.27; *P* = 0.003). Similar results were found in men and participants < 60 years. The Kaplan-Meier analysis demonstrated a significant increase in cancer occurrence in participants with a baPWV ≥ 18 m/s (*P* < 0.001) (Figures 3A–C). However, women and participants ≥ 60 years had a similar risk of cancer across different categories of baPWV, and the Kaplan Meier curves were shown in Supplementary Figures 2A, B.

**TABLE 2:** Adjusted hazard ratios (95% CI) of cancer according to brachial-ankle pulse wave velocity (baPWV).

Subgroups	baPWV (m/s)			*P*-value	Per 1SD increase in baPWV	*P*-value
	**<14.0**	**14.0–18.0**	**≥18.0**			
Entire cohort (*n* = 45,627)	Ref.	1.16 (0.92, 1.46)	1.51 (1.14, 2.00)	0.004	1.16 (1.05, 1.27)	0.003
Age < 60 years (*n* = 36,872)	Ref.	1.22 (0.94, 1.59)	1.75 (1.20, 2.55)	0.006	1.30 (1.13, 1.49)	0.002
Age ≥ 60 years (*n* = 8,755)	Ref.	1.04 (0.61, 1.79)	1.28 (0.74, 2.20)	0.165	1.08 (0.95, 1.22)	0.274
Men (*n* = 32,847)	Ref.	1.26 (0.94, 1.69)	1.72 (1.22, 2.43)	0.002	1.17 (1.05, 1.31)	0.004
Women (*n* = 12,780)	Ref.	1.13 (0.77, 1.64)	1.26 (0.73, 2.17)	0.391	1.16 (0.96, 1.39)	0.125

Adjusted for age, sex, anemia, MAP, body mass index, hs-CRP, total cholesterol, fasting plasma glucose, uric acid, eGFR, current smoker, current drinker, high/intensive activity, hepatic dysfunction, antihypertensive use, HBsAg positive, and tumor family history. 1 SD of baPWV: 3.52 m/s.

### 3.4. Risk of cancer at different systems based on different categories of baPWV

Participants with increased baPWV had a higher likelihood of being diagnosed with digestive cancer compared to those in the groups of lower baPWV (Table 3). The HR and 95% CI for digestive cancer across moderate (baPWV = 14.0∼18.0 m/s) and high (baPWV ≥ 18 m/s) levels of arterial stiffness compared to the patients with normal elasticity were 1.55 (1.00∼2.40), and 1.99 (1.19∼3.33) (*P* for trend = 0.010). The risk of digestive cancer according to Kaplan-Meir analysis based on the baPWV is described in Figure 3D. However, participants with increased baPWV had a similar likelihood of being diagnosed as cancer of respiratory, urogenital, or other systems across different categories of baPWV, and the Kaplan Meier curves were shown in Supplementary Figures 2C–E.

**TABLE 3:** Adjusted hazard ratios associated with cancer of different systems.

Subgroups	baPWV (m/s)			*P*-value	Per 1SD increase in baPWV	*P*-value
	**<14.0**	**14.0–18.0**	**≥18.0**			
Digestive system (*n* = 170)	Ref.	1.55 (1.00, 2.40)	1.99 (1.19, 3.33)	0.010	1.30 (1.12, 1.51)	0.001
Respiratory system (*n* = 138)	Ref.	1.15 (1.72, 1.84)	1.70 (0.97, 2.95)	0.055	1.11 (0.93, 1.34)	0.254
Urogenital system (*n* = 89)	Ref.	0.82 (0.46, 1.46)	0.85 (0.42, 1.74)	0.670	0.91 (0.71, 1.18)	0.470
Other systems (*n* = 156)	Ref.	1.16 (0.78, 1.74)	1.51 (0.86, 2.66)	0.166	1.19 (0.97, 1.46)	0.088

Adjusted for age, sex, anemia, MAP, body mass index, hs-CRP, total cholesterol, fasting plasma glucose, uric acid, eGFR, current smoker, current drinker, high/intensive activity, hepatic dysfunction, antihypertensive use, HBsAg positive, and tumor family history. 1 SD of baPWV: 3.52 m/s.

### 3.5. Restricted spline curve analysis

**FIGURE 4:**
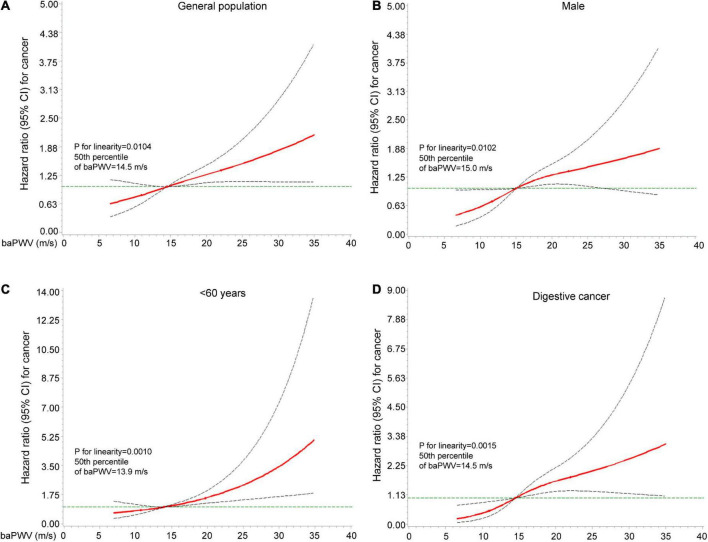
Risk of cancer according to restricted spline curve. Spline-curve analyses show a linearly increasing risk, linking the severity of arterial stiffness to overall cancer risk in the entire cohort **(A)** men **(B)** and participants < 60-year-old **(C)**. Spline-curve analyses show a linearly increasing risk, linking the severity of arterial stiffness to digestive cancer risk in the entire cohort **(D)**. Hazard ratios of restricted cubic spline transformation of baPWV with 3 knots (5, 50, and 95% of baPWV value) and 50% of baPWV value as a reference are plotted. Red lines are hazard ratios and gray lines are 95% confidence intervals. baPWV, Brachial-ankle pulse wave velocity.

When baPWV (restricted cubic splines with 3 knots at 5, 50, and 95%) were computed, the observed associations with high baPWV were generally unchanged (Figure 4). Spline-curve analyses showed a linearly increasing risk, linking the severity of arterial stiffness to overall cancer risk in the entire cohort (*P* = 0.0104), men (*P* = 0.0102), and participants < 60 years old (*P* = 0.0010). There is also a significant relationship between baPWV and the risk of digestive cancer (*P* = 0.0015). However, there is no significant relationship between baPWV and the risk of respiratory (*P* = 0.2554), urogenital (*P* = 0.7671), and other malignancies (*P* = 0.1110).

### 3.6. Sensitivity analyses

**FIGURE 5:**
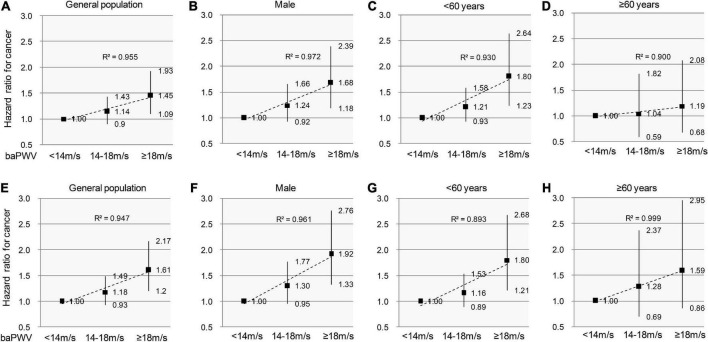
Sensitivity analyses to test the association between arterial stiffness and incident cancer in individuals without PAD/CVDs. Participants are categorized into 3 groups according to baPWV values. The sign of PAD is defined as ABI ≤ 0.9 and/or IAD ≥ 15 mmHg. The hazard ratios and 95% confidence intervals for cancer incidence remain essentially unchanged after excluding the individuals with PAD/CVDs in the entire cohort **(A,E)**, men **(B,F)**, individuals < 60-year-old **(C,G)**, and individuals ≥ 60-year-old **(D,H)**. The dashed trend lines fit HRs well and R^2^ show a linearly increasing risk for cancer incidence. PAD, Peripheral arterial disease; CVDs, cardiovascular diseases; baPWV, brachial-ankle pulse wave velocity.

Pulse wave velocity increases with peripheral arterial diseases and severe cardiovascular disease (CVDs) conditions. As such, we performed a sensitivity analysis to control the effect of severe vascular diseases. The analysis was repeated to test for the robustness of estimates in those who had no history of CVDs (*n* = 43,900) or had no evidence of peripheral arterial diseases (*n* = 41,511). These results remained essentially unchanged after adjustment using the clinical confounder model (Figure 5).

## 4. Discussion

The results of this large-scale observational study unveiled a positive association between arterial stiffness and cancer. The incidence density of cancer was increased with heightened arterial stiffness in men and the younger participants. Also, arterial stiffness measured by baPWV was associated with digestive cancer.

To the extent of our knowledge, this was the first study to evaluate the association between baPWV and malignant disease. In the past, several studies demonstrated an association between arterial stiffness and CVDs (23–25) during/after chemotherapy. However, there was a lack of robust knowledge on whether arterial stiffness by itself is sufficient to promote cancer severity and outcomes. The findings of the present study could be majorly attributed to: (i) the intimate relationship between CVDs and new-onset cancers, which may result from several shared biological mechanisms such as inflammation and reactive oxygen species (26).

The association of arterial stiffness and cancer events was found to differ in sex. Our findings support that high baPWV is associated with cancer incidence in men, but not in women. This discrepancy could be explained by the difference in the prevalence of hypertension between men and women, which is higher in men (27), and its associated adverse cardiovascular outcomes (28). A previous study reported a higher risk of cancer in the hypertension group compared to non-hypertension patients (29). Furthermore, Jessica et al. found that subjects with SBP 160∼179 mmHg or DBP 100∼10 mmHg had an 8% increased risk for cancer incidence compared with the lowest grade of hypertension (12).

The baPWV was also significantly associated with the risk of cancer in participants younger than 60 years, whereas this association was weakened to a non-significant level when we analyzed data in the participants ≥ 60 years. baPWV is known to be positively associated with age (30). Of the many risk factors associated with baPWV, age and blood pressure are by far the most important factors influencing baPWV parameters (31–33). Aging *per se* is a promoter of arterial stiffness, especially in the older population (15, 33, 34). Consequently, this may mask the effect of baPWV for cancer in the age category ≥ 60 years, but not in participants < 60 years. Therefore, further follow-up studies are required to validate the potential benefit of implementing baPWV in the screening model in the middle-aged population before the development of cancer.

Despite the risk of cardiovascular disease increasing linearly with the increase in baPWV (10), the results of the present study remain essentially unchanged after excluding the effect of severe vascular diseases. The data strongly indicates the presence of an association between arterial stiffness and the progression of cancer. This implies the possibility of defining risk thresholds by baPWV in the future, specifically tailored to the contemporary concept of Onco-cardiology, to improve risk stratification with established grading algorithms.

The combination of the aforementioned commonly shared risk factors and inflammation can contribute to the pathogenesis of artery rigidity. Consequently, arterial stiffness would be accelerated. But that doesn’t fully explain how increased arterial stiffness is associated with cancer. Although it is beyond the scope of this study to investigate the biological mechanisms underlying the association between arterial stiffness and cancer events, we can speculate that both cancer (35) and arterial dysfunction (36) are characterized by inflammation. Likewise, CVD and cancer share inflammation and ROS as common biological mechanisms (26). Moreover, the alterations in the levels of secreted factors that regulate the prognosis of CVDs and malignancy may play a vital role in the communication between the cardiovascular system and tumors in the body (37, 38).

To the extent of our knowledge, this is the first major study to recognize the prognostic implications of baPWV and the incidence of cancer. However, this study must be interpreted in the context of some significant limitations. Arterial stiffness as a marker of vascular dysfunction is an operator-dependent procedure. The unavailability of HbA1C records for many patients limited us from considering it in our analysis. Our study only registered the Chinese population living in Northern China, which may limit the generalization of our results. However, the homogeneous nature of our prospective study provided absolute control of potential confounding effects that could result from racial disparities and health service inequalities. Therefore, the enrolment of only Chinese ethnicity in this study could bolster its internal validity. Cancer itself can affect systemic vascular function, thus multiple baPWV examinations during follow-up would benefit the study. The increased cancer risk by ∼50% was not justified by the slightly increased incidence of cancer across the corresponding baPWV groups. Thus, confounding factors could have strongly influenced the results of the present study. Also, identifying the time (in days or months) of a final baPWV measurement prior to cancer diagnosis would have added significant value to the clinical research. Therefore, further research is needed to confirm whether this association exists among other ethnic groups. Of note, possible categorization of tumors might be more helpful since for example tumors on solid organs such as respiratory cancer show a trend but perhaps due to the small number is not becoming statistically significant. Moreover, the concept of vascular aging might be relevant since the prediction is better at younger ages with stiffer arteries implying early vascular aging. Hence, a possible analysis based on expected values of vascular aging in solid tumors will be a benefit in confirming the association between baPWV and cancer incidence.

## 5. Conclusion

In conclusion, the results of this study demonstrate a positive association between high baPWV and cancer occurrence, with a potentially novel correlation. The findings of our study further recommend that individuals should engage in healthy lifestyle practices to maintain a lower risk of developing arterial stiffness. Further study is required to confirm the observed associations between arterial stenosis and the occurrence of cancer.

## Data availability statement

The raw data supporting the conclusions of this article will be made available by the authors, without undue reservation.

## Author contributions

YJ and XY: conceptualization. TH: data curation. AX: formal analysis and investigation. Y-LX: funding acquisition and validation. TH, JL, and SC: methodology. SW: project administration. YJ: writing—original draft. All authors contributed to the article and approved the submitted version.

## References

[1] Mozos I Borzak G Caraba A Mihaescu R. Arterial stiffness in hematologic malignancies. *Onco Targets Ther.* (2017) 10:1381–8. 10.2147/OTT.S126852 28424554 PMC5344421

[2] Parr S Liang J Schadler K Gilchrist S Steele C Ade C. Anticancer therapy-related increases in arterial stiffness: a systematic review and meta-analysis. *J Am Heart Assoc.* (2020) 9:e015598. 10.1161/JAHA.119.015598 32648507 PMC7660726

[3] Zamorano J Lancellotti P Rodriguez Muñoz D Aboyans V Asteggiano R Galderisi M 2016 ESC posi-tion paper on cancer treatments and cardiovascular toxicity developed under the auspices of the ESC commit-tee for practice guidelines: the task force for cancer treatments and cardiovascular toxicity of the European Society of Cardiology (ESC). *Eur Heart J.* (2016) 37:2768–801. 10.1093/eurheartj/ehw211 27567406

[4] Meijers W de Boer R. Common risk factors for heart failure and cancer. *Cardiovasc Res.* (2019) 115:844–53. 10.1093/cvr/cvz035 30715247 PMC6452432

[5] Hasin T Gerber Y Weston S Jiang R Killian J Manemann S Heart failure after myocardi-al infarction is associated with increased risk of cancer. *J Am Coll Cardiol.* (2016) 68:265–71. 10.1016/j.jacc.2016.04.053 27417004 PMC4947209

[6] Hasin T Gerber Y McNallan S Weston S Kushwaha S Nelson T Patients with heart failure have an increased risk of incident cancer. *J Am Coll Cardiol.* (2013) 62:881–6. 10.1016/j.jacc.2013.04.088 23810869 PMC3758775

[7] Banke A Schou M Videbaek L Møller J Torp-Pedersen C Gustafsson F Incidence of cancer in patients with chronic heart failure: a long-term follow-up study. *Eur J Heart Fail.* (2016) 18:260–6. 10.1002/ejhf.472 26751260

[8] Sung H Ferlay J Siegel R Laversanne M Soerjomataram I Jemal A Global cancer statistics 2020: GLO-BOCAN estimates of incidence and mortality worldwide for 36 cancers in 185 countries. *CA Cancer J Clin.* (2021) 71:209–49. 10.3322/caac.21660 33538338

[9] Cao Z Xu C Yang H Li S Wang Y. The role of healthy lifestyle in cancer incidence and temporal transitions to cardiometabolic disease. *JACC CardioOncol.* (2021) 3:663–74. 10.1016/j.jaccao.2021.09.016 34988474 PMC8702801

[10] Tanaka A Tomiyama H Maruhashi T Matsuzawa Y Miyoshi T Kabutoya T Physiological diagnostic criteria for vascular failure. *Hypertension.* (2018) 72:1060–71. 10.1161/HYPERTENSIONAHA.118.11554 30354826

[11] Aroor A Jia G Sowers J. Cellular mechanisms underlying obesity-induced arterial stiffness. *Am J Physiol Regul Integr Comp Physiol.* (2018) 314:R387–98. 10.1152/ajpregu.00235.2016 29167167 PMC5899249

[12] Harding J Sooriyakumaran M Anstey K Adams R Balkau B Brennan-Olsen S Hypertension, antihy-pertensive treatment and cancer incidence and mortality: a pooled collaborative analysis of 12 Australian and New Zealand cohorts. *J Hypertens.* (2016) 34:149–55. 10.1097/HJH.0000000000000770 26630217

[13] Lerman L Kurtz T Touyz R Ellison D Chade A Crowley S Animal models of hypertension: a scientific statement from the American Heart Association. *Hypertension.* (2019) 73:e87–120. 10.1161/HYP.0000000000000090 30866654 PMC6740245

[14] Avraham S Abu-Sharki S Shofti R Haas T Korin B Kalfon R Early cardiac remodeling promotes tumor growth and metastasis. *Circulation.* (2020) 142:670–83. 10.1161/CIRCULATIONAHA.120.046471 32475164

[15] Munakata M. Brachial-ankle pulse wave velocity in the measurement of arterial stiffness: recent evidence and clinical applications. *Curr Hypertens Rev.* (2014) 10:49–57. 10.2174/157340211001141111160957 25392144

[16] Wu S Jin C Li S Zheng X Zhang X Cui L Aging, arterial stiffness, and blood pressure association in Chinese adults. *Hypertension.* (2019) 73:893–9. 10.1161/HYPERTENSIONAHA.118.12396 30776974

[17] Chen S Li W Jin C Vaidya A Gao J Yang H Resting heart rate trajectory pattern predicts arterial stiff-ness in a community-based Chinese cohort. *Arterioscler Thromb Vasc Biol.* (2017) 37:359–64. 10.1161/ATVBAHA.116.308674 27908892 PMC5269420

[18] Miyashima M Shoji T Kakutani Y Yamazaki Y Ochi A Morioka T Inter-arm blood pressure difference in diabetes mellitus and its preferential association with peripheral artery disease. *J Atheroscler Thromb.* (2020) 27:780–8. 10.5551/jat.52886 31813900 PMC7458791

[19] Williams B Mancia G Spiering W Agabiti Rosei E Azizi M Burnier M 2018 ESC/ESH guidelines for the management of arterial hypertension. *Eur Heart J.* (2018) 39:3021–104. 10.1097/HJH.0000000000001940 30165516

[20] Singal A Bataller R Ahn J Kamath P Shah V. ACG clinical guideline: alcoholic liver disease. *Am J Gastroenterol.* (2018) 113:175–94. 10.1038/ajg.2017.469 29336434 PMC6524956

[21] Sarin S Choudhury A Sharma M Maiwall R Al Mahtab M Rahman S Acute-on-chronic liver failure: consensus recommendations of the Asian Pacific association for the study of the liver (APASL): an update. *Hepatol Int.* (2019) 13:353–90. 31172417 10.1007/s12072-019-09946-3 PMC6728300

[22] Yoshiji H Nagoshi S Akahane T Asaoka Y Ueno Y Ogawa K Evidence-based clinical practice guidelines for liver cirrhosis 2020. *J Gastroenterol.* (2021) 56:593–619. 10.1007/s00535-021-01788-x 34231046 PMC8280040

[23] Tomiyama H Matsumoto C Shiina K Yamashina A. Brachial-ankle PWV: current status and future direc-tions as a useful marker in the management of cardiovascular disease and/or cardiovascular risk factors. *J Atheroscler Thromb.* (2016) 23:128–46. 10.5551/jat.32979 26558401

[24] Koji Y Tomiyama H Ichihashi H Nagae T Tanaka N Takazawa K Comparison of ankle-brachial pres-sure index and pulse wave velocity as markers of the presence of coronary artery disease in subjects with a high risk of atherosclerotic cardiovascular disease. *Am J Cardiol.* (2004) 94:868–72. 10.1016/j.amjcard.2004.06.020 15464667

[25] Chung C Tseng Y Lin Y Hsu J Wang P. Association of brachial-ankle pulse wave velocity with ather-osclerosis and presence of coronary artery disease in older patients. *Clin Interv Aging.* (2015) 10:1369–75. 10.2147/CIA.S89568 26316732 PMC4548723

[26] Koene R Prizment A Blaes A Konety S. Shared risk factors in cardiovascular disease and cancer. *Circulation.* (2016) 133:1104–14. 10.1161/CIRCULATIONAHA.115.020406 26976915 PMC4800750

[27] Wang Z Chen Z Zhang L Wang X Hao G Zhang Z Status of hypertension in China: results from the China hypertension survey, 2012–2015. *Circulation.* (2018) 137:2344–56. 10.1161/CIRCULATIONAHA.117.032380 29449338

[28] Mozaffarian D Benjamin E Go A Arnett D Blaha M Cushman M Executive summary: heart disease and stroke statistics–2016 update: a report from the American Heart Association. *Circulation.* (2016) 133:447–54. 10.1161/CIR.0000000000000366 26811276

[29] Raynor W Jr Shekelle R Rossof A Maliza C Paul O. High blood pressure and 17-year cancer mortality in the Western Electric Health Study. *Am J Epidemiol.* (1981) 113:371–7. 10.1093/oxfordjournals.aje.a113105 7211822

[30] Avolio A Kuznetsova T Heyndrickx G Kerkhof P Li J. Arterial flow, pulse pressure and pulse wave velocity in men and women at various ages. *Adv Exp Med Biol.* (2018) 1065:153–68. 30051383 10.1007/978-3-319-77932-4_10

[31] Baier D Teren A Wirkner K Loeffler M Scholz M. Parameters of pulse wave velocity: determinants and refer-ence values assessed in the population-based study LIFE-adult. *Clin Res Cardiol.* (2018) 107:1050–61. 10.1007/s00392-018-1278-3 29766282 PMC6208658

[32] Dumor K Shoemaker-Moyle M Nistala R Whaley-Connell A. Arterial stiffness in hypertension: an update. *Curr Hypertens Rep.* (2018) 20:72. 10.1007/s11906-018-0867-x 29974262

[33] AlGhatrif M Wang M Fedorova O Bagrov A Lakatta E. The pressure of aging. *Med Clin North Am.* (2017) 101:81–101. 10.1016/j.mcna.2016.08.006 27884238 PMC5206899

[34] Cecelja M Chowienczyk P. Dissociation of aortic pulse wave velocity with risk factors for cardiovascular dis-ease other than hypertension: a systematic review. *Hypertension.* (2009) 54:1328–36. 10.1161/HYPERTENSIONAHA.109.137653 19884567

[35] Diakos C Charles K McMillan D Clarke S. Cancer-related inflammation and treatment effectiveness. *Lancet Oncol.* (2014) 15:e493–503. 10.1016/S1470-2045(14)70263-3 25281468

[36] Geovanini G Libby P. Atherosclerosis and inflammation: overview and updates. *Clin Sci.* (2018) 132:1243–52. 10.1042/CS20180306 29930142

[37] Daughaday W Deuel T. Tumor secretion of growth factors. *Endocrinol Metab Clin North Am.* (1991) 20:539–63. 10.1016/S0889-8529(18)30258-5 1718747

[38] Fountoulaki K Dagres N Iliodromitis E. Cellular communications in the heart. *Card Fail Rev.* (2015) 1:64–8. 10.15420/cfr.2015.1.2.64 28785434 PMC5490974

