# Supplementary material for: The association between arterial stiffness and cancer occurrence: Data from Kailuan cohort study
Source: Front Cardiovasc Med. 2023 Mar 1;10:1112047. doi: 10.3389/fcvm.2023.1112047 (PMC10014543; doi:10.3389/fcvm.2023.1112047)
Supplement: Supplementary file 1 [file Table_1.DOCX]

Supplementary Material

**Supplemental Table S1.** Univariate and multivariate Cox regression analysis of cancer occurrence.

| Variable | Univariate analysis | | Multivariate analysis | |
| --- | --- | --- | --- | --- |
|  | HR（95%CI） | P value | HR（95%CI） | P value |
| baPWV 14-18(m/s) | 1.39(1.14,1.70) | 0.001 | 1.16(0.92,1.46) | 0.201 |
| baPWV≥18(m/s) | 2.45(1.98,3.04) | <0.001 | 1.51(1.14,2.00) | 0.004 |
| Age (year) | 2.58(2.17,3.06) | <0.001 | 2.27(1.84,2.80) | <0.001 |
| Male | 1.89(1.74,1.06) | <0.001 | 1.67(1.54,1.82) | <0.001 |
| SBP (mmHg) | 1.01 (1.01,1.02) | 0.001 | 1.00(1.00,1.01) | 0.032 |
| DBP (mmHg) | 1.01(1.00,1.02) | 0.032 | 1.00(0.99,1.01) | 0.587 |
| MAP (mmHg) | 1.01(1.01,1.02) | <0.0001 | 1.01(1.00,1.01) | 0.245 |
| BMI (kg/m^2^) | 1.20(1.01,1.42) | 0.039 | 1.14(0.95,1.36) | 0.149 |
| hs-CRP>2mg/L | 1.36(1.13,1.63) | 0.001 | 1.17(0.93,1.47) | 0.370 |
| TC (mmol/L) | 1.01(0.97,1.06) | 0.600 | 0.98(0.92,1.05) | 0.517 |
| HDL (mmol/L) | 1.03(0.96,1.12) | 0.407 | 1.03(0.95,1.12) | 0.501 |
| FPG (mmol/L) | 1.07(1.03,1.12) | 0.001 | 1.01(0.97,1.07) | 0.586 |
| Uric acid (mmol/L) | 0.92(0.71,1.20) | 0.547 | 0.88(0.68,1.15) | 0.349 |
| eGFR (mL/min*1.73m^2^) | 1.20(0.91,1.58) | 0.210 | 1.05(0.78,1.40) | 0.761 |
| Current smoker | 1.39(1.23,1.68) | 0.892 | 1.25(1.05,1.55) | 0.039 |
| Current drinker | 1.01(0.85,1.21) | 0.672 | 1.39(1.02,1.90) | 0.037 |
| High/intensive activity | 1.24(0.75,1.31) | 0.563 | 1.11(0.93,1.32) | 0.248 |
| Hepatic dysfunction | 0.97(0.71,1.32) | 0.820 | 1.02(0.74,1.41) | 0.903 |
| Anemia | 1.25(0.88,1.76) | 0.215 | 1.52(1.05,2.20) | 0.027 |
| HBsAg positive | 2.44(1.54,3.86) | <0.001 | 2.53(1.60, 4.01) | <0.001 |
| Antihypertensive use | 1.60(1.31,1.94) | <0.001 | 1.03(0.83, 1.29) | 0.779 |
| Tumor family history | 1.15(0.84,1.58) | 0.392 | 0.88(0.63, 1.22) | 0.432 |

Adjusted for age, sex, anemia, MAP, body mass index, hs-CRP, total cholesterol, fasting plasma glucose, uric acid, eGFR, current smoker, current drinker, high/intensive activity, hepatic dysfunction, antihypertensive use, HBsAg positive, and tumor family history.

**Supplemental Table S2. Cumulative incidence of cancer in participants by groups of baPWV (%)**

|  | baPWV <14.0  m/s (n=19679) | 14.0≤ baPWV <18.0 m/s (n=17720) | baPWV ≥18.0  m/s (n=8228) | χ^2^ | *P-*value |
| --- | --- | --- | --- | --- | --- |
| General population | 1.80 | 2.24 | 3.83 | 73.69 | <0.001 |
| Age <60 years | 1.74 | 1.67 | 2.55 | 12.05 | 0.002 |
| Age ≥60 years | 3.45 | 4.16 | 4.80 | 2.88 | 0.237 |
| Male | 1.45 | 2.20 | 3.86 | 65.94 | <0.001 |
| Female | 2.11 | 2.59 | 3.86 | 16.84 | <0.001 |
| Digestive system | 0.39 | 0.75 | 1.45 | 55.41 | <0.001 |
| Respiratory system | 0.40 | 0.55 | 1.18 | 38.00 | <0.001 |
| Urogenital system | 0.35 | 0.38 | 0.50 | 8.19 | 0.017 |
| Other systems | 0.68 | 0.66 | 0.62 | 0.71 | 0.702 |

**Supplemental Table S3. Incidence and crude hazard ratios for cancer**

|  | **Number**  **(n)** | **Follow-up time (person-years)** | **Incidence Density (per 10,000 person-years)** | **Crude HR**  **(95% CI)** | ***P-*value** |
| --- | --- | --- | --- | --- | --- |
| **General population (n=45627)** | | | | | |
| <14.0 | 172 | 75590.58 | 2.28 | Ref. |  |
| 14.0-18.0 | 212 | 66924.65 | 3.17 | 1.39(1.14,1.70) | 0.001 |
| ≥18.0 | 169 | 30260.47 | 5.58 | 2.45(1.98,3.04) | <0.0001 |
| per 1SD (3.52m/s) increase | | |  | 1.36(1.27,1.45) | <0.0001 |
| **Age <60 years (n=36872)** | | | | | |
| <14.0 | 156 | 72528.21 | 2.15 | Ref. |  |
| 14.0-18.0 | 132 | 52899.08 | 2.50 | 1.15(0.91,1.45) | 0.232 |
| ≥18.0 | 51 | 13434.15 | 3.80 | 1.74(1.27,2.39) | 0.0001 |
| per 1SD (3.52m/s) increase | | |  | 1.25(1.12,1.41) | 0.0001 |
| **Age ≥60 years (n=8755)** | | | | | |
| <14.0 | 16 | 3062.37 | 5.22 | Ref. |  |
| 14.0-18.0 | 80 | 14025.56 | 5.70 | 1.10(0.64,1.88) | 0.728 |
| ≥18.0 | 118 | 16826.31 | 7.01 | 1.36(0.81,2.29) | 0.249 |
| per 1SD (3.52m/s) increase | | |  | 1.08(0.96,1.22) | 0.186 |
| **Male (n=32847)** | | | | | |
| <14.0 | 74 | 41290.75 | 1.79 | Ref. |  |
| 14.0-18.0 | 151 | 52077.56 | 2.90 | 1.60(1.21,2.11) | 0.001 |
| ≥18.0 | 131 | 23895.94 | 5.48 | 3.01(2.26,4.00) | <0.0001 |
| per 1SD (3.52m/s) increase | | |  | 1.42(0.30,1.54) | <0.0001 |
| **Female (n=12780)** | | | | | |
| <14.0 | 98 | 34299.83 | 2.85 | Ref. |  |
| 14.0-18.0 | 61 | 14847.09 | 4.07 | 1.44(1.05,1.99) | 0.025 |
| ≥18.0 | 38 | 6364.53 | 5.80 | 2.11(1.45,3.07) | <0.0001 |
| per 1SD (3.52m/s) increase | | |  | 1.34(1.19,1.51) | <0.0001 |

**
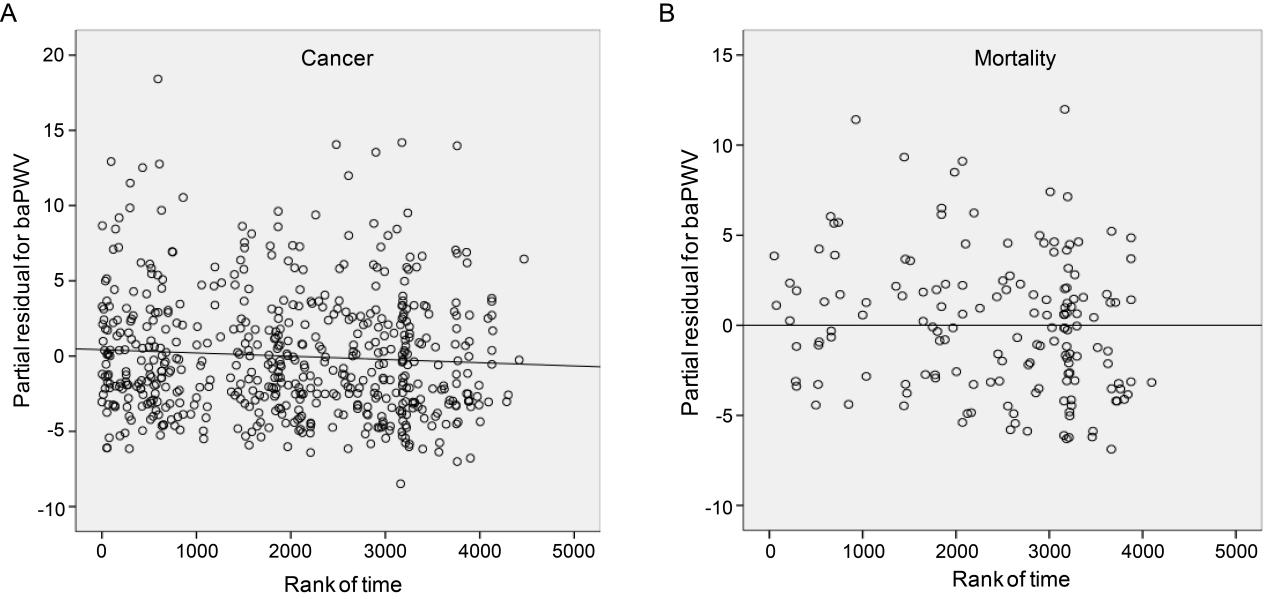
**

**Supplemental Figure 1.** **The smoothing curve of Schoenfeld partial residuals against time rank.** The smoothing curve of cancer incidence (A) and all-cause mortality in cancer patients (B) in all participants.

**
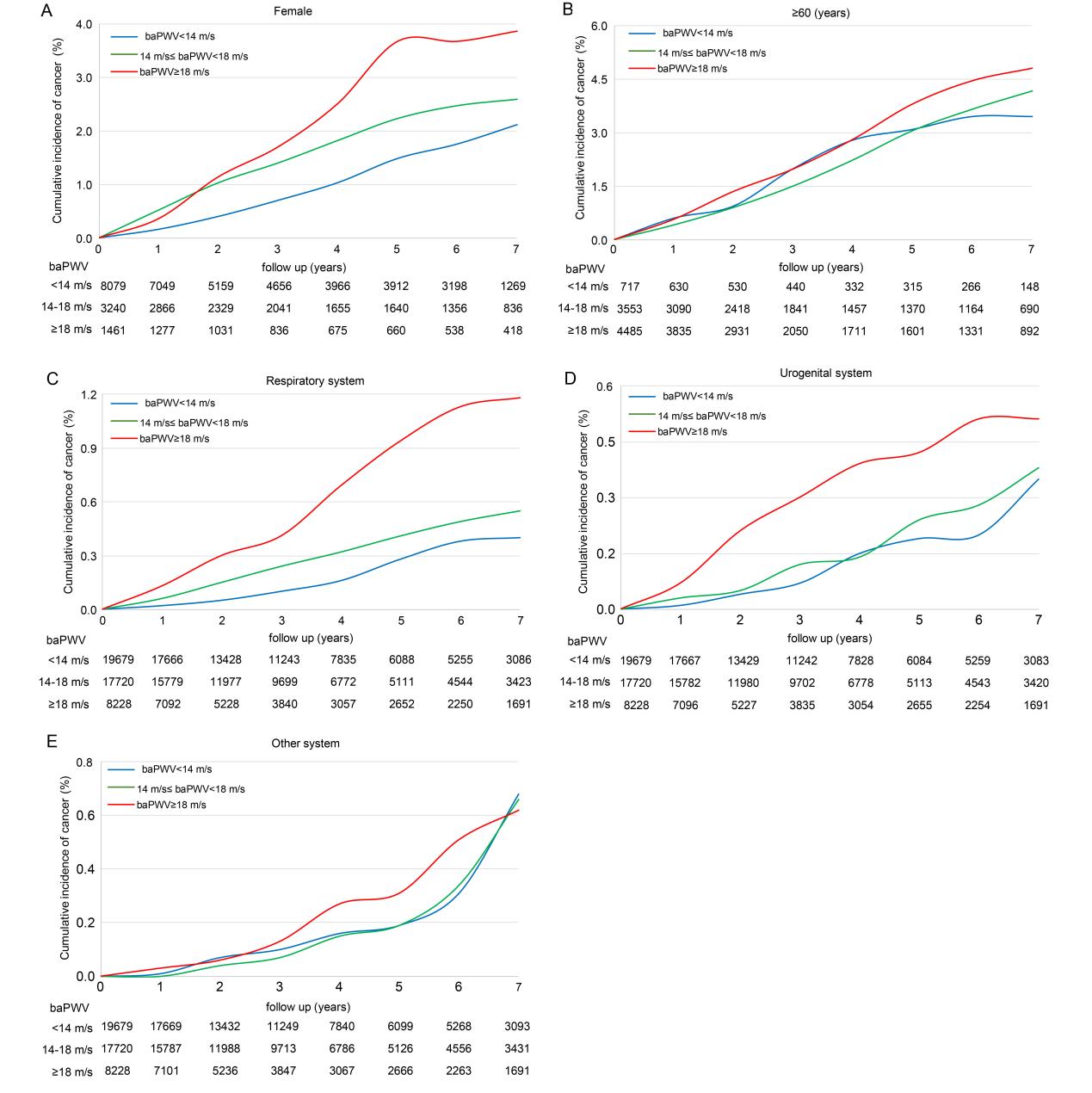
**

**Supplemental Figure 2.** **The Kaplan–Meier survival curves for cancer incidence.** The risk of cancer occurrence according to Kaplan-Meir analysis in females (A), participants >60 years (B), respiratory system (C) urogenital system (D), and other system (E).
